# DNA methylation profiles of bronchoscopic biopsies for the diagnosis of lung cancer

**DOI:** 10.1186/s13148-021-01024-6

**Published:** 2021-02-17

**Authors:** Torsten Goldmann, Bernhard Schmitt, Julia Müller, Maren Kröger, Swetlana Scheufele, Sebastian Marwitz, Dörte Nitschkowski, Marc A. Schneider, Michael Meister, Thomas Muley, Michael Thomas, Christian Kugler, Klaus F. Rabe, Reiner Siebert, Martin Reck, Ole Ammerpohl

**Affiliations:** 1 Pathology of the University Medical Center Schleswig-Holstein (UKSH), Campus Lübeck and the Research Center Borstel, Lübeck, Borstel, Germany; 2 Labor Lademannbogen MVZ GmbH, Hamburg, Germany; 3 Institute of Human Genetics, University Medical Center Schleswig-Holstein (UKSH), Campus Kiel, Germany; 4 LungenClinic Grosshansdorf, Grosshansdorf, Germany; 5 Translational Research Unit, Thoraxklinik at University Hospital Heidelberg, 69126 Heidelberg, Germany; 6 Translational Lung Research Center Heidelberg (TLRC), German Center for Lung Research (DZL), Heidelberg, Germany; 7 Internistische Onkologie der Thoraxtumoren, Thoraxklinik im Universitätsklinikum Heidelberg, Translational Lung Research Center Heidelberg (TLRC-H), Member of the German Center for Lung Research (DZL), Heidelberg, Germany; 8 Institute of Human Genetics, University Medical Center Ulm, Albert-Einstein-Allee 11, 89081 Ulm, Germany; 9 Airway Research Center North, Member of the German Center for Lung Research (DZL), Grosshansdorf, Germany

**Keywords:** Lung cancer, DNA methylation, Paired biopsies

## Abstract

**Background:**

Lung cancer is the leading cause of cancer-related death in most western countries in both, males and females, accounting for roughly 20–25% of all cancer deaths. For choosing the most appropriate therapy regimen a definite diagnosis is a prerequisite. However, histological characterization of bronchoscopic biopsies particularly with low tumor cell content is often challenging. Therefore, this study aims at (a) determining the value of DNA methylation analysis applied to specimens obtained by bronchoscopic biopsy for the diagnosis of lung cancer and (b) at comparing aberrantly CpG loci identified in bronchoscopic biopsy with those identified by analyzing surgical specimens.

**Results:**

We report the HumanMethylation450-based DNA methylation analysis of paired samples of bronchoscopic biopsy specimens either from the tumor side or from the contralateral tumor-free bronchus in 37 patients with definite lung cancer diagnosis and 18 patients with suspicious diagnosis. A differential DNA methylation analysis between both biopsy sites of patients with definite diagnosis identified 1303 loci. Even those samples were separated by the set of 1303 loci in which histopathological analysis could not unambiguously define the dignity. Further differential DNA methylation analyses distinguished between SCLC and NSCLC. We validated our results in an independent cohort of 40 primary lung cancers obtained by open surgical resection and their corresponding controls from the same patient as well as in publically available DNA methylation data from a TCGA cohort which could also be classified with high accuracy.

**Conclusions:**

Considering that the prognosis correlates with tumor stage at time of diagnosis, early detection of lung cancer is vital and DNA methylation analysis might add valuable information to reliably characterize lung cancer even in histologically ambiguous sample material.

**Supplementary Information:**

The online version contains supplementary material available at 10.1186/s13148-021-01024-6.

## Background

In oncology, a reliable diagnosis of a cancer and a definite differentiation between benign and malignant processes is a prerequisite for choosing the most appropriate therapy modality. In the suspicion of lung cancer, it is regularly attempted to confirm the diagnosis from one or more biopsies obtained by bronchoscopy. However, histological characterization of bronchoscopic biopsies particularly of early tumor stages with low tumor cell content and putatively altered histology e.g. due to other precursor lesions or inflammatory processes is often challenging. Therefore, additional tools to support detection and diagnosis of lung cancer are desirable.

DNA methylation is an epigenetic modification of the DNA which is mandatory for regulating and adapting gene activity as well as for differentiation and development [1, 2]. Aberrant DNA methylation patterns are characteristic for both hematopoietic malignancies as well as solid tumors, including lung cancer [3]. Several research groups and international consortia (e.g. TCGA) have characterized epigenetic alterations in lung cancer in detail [4]. Some authors also speculated about the putative application of these data to develop tools such as DNA methylation-based panels for diagnostic purposes [5]. Nevertheless, most of these analyses have been performed using surgically resected lung cancer specimens of high quality and/or high tumor cell content. Though the use of such surgically resected samples of high purity is rational for understanding the biology of malignant cells and, in particular, for identifying putative therapeutic targets, the samples do not necessarily reflect the situation in initial diagnostics. The histology of biopsy specimens e.g. collected during bronchoscopy, is often more difficult to assess as compared to primary tumor specimens. This is reflected e.g. in a lower overall amount of available tissue and/or a lower tumor cell content, which both can render detection and characterization of tumor cells sometimes challenging. On the other hand, for the detection of a malignancy aberrant DNA methylation patterns of microenvironmental cells might also be considered valuable for diagnosis—as long as these are characteristic for the malignant tumor. Indeed, epigenetic alterations in non-malignant cells of the microenvironment have been described before [6]. Consequently, the sample material used for building a classifier should be considered thoroughly in advance.

In order to address these particularities inherent to bronchoscopic biopsies as compared to primary lung cancers, this study focuses on characterizing altered DNA methylation patterns in tumor-containing as compared to matched normal specimens collected during bronchoscopy also available to pathologists for performing diagnosis. The study also included bronchoscopic samples in which it was difficult by conventional pathology to reach a diagnosis. Moreover, the results were compared with data collected from an independent set of high quality specimens of surgically resected primary tumors. These latter samples were subjected to macrodissection to further increase the tumor cell fraction to identify DNA methylation patterns characteristic or common for both kinds of specimens. Additionally, a common set of aberrantly methylated loci has been identified. We speculate that the analysis of such a set of loci might supplement diagnostics of challenging cases of lung cancer in future. Nevertheless, this study does not aim at establishing a distinct clinical biomarker panel.

## Results

To identify recurrent epigenetic alterations in lung cancer and to reveal their putative benefit for diagnostics, HumanMethylation450 BeadChip analyses were performed on biopsy samples collected during bronchoscopy (further called: "biopsy samples"), lung cancer specimens of surgically resected primary tumors (further called "surgical specimens") and corresponding normal lung control specimens collected from the same patients (Additional file 2: Fig. S1).

While surgically resected tumor specimens usually contain plenty of tumor cells allowing a reliable histological diagnosis, analyses of biopsy samples more often suffer from low sample quality, e.g. due to low tumor cell content or putatively altered histology, making diagnosis more difficult and error-prone. To address the question whether DNA methylation analysis might add reliability to classical diagnostics of demanding cases, paired biopsy samples (i.e. from a supposedly tumor-containing and a supposedly tumor free contralateral bronchus) were collected from 55 patients during bronchoscopy. All samples were subsequently surveyed by experienced lung pathologists using standard histopathological procedures (Additional file 6: Table S1). In a total of 74 of the 110 biopsies, definite histopathological diagnosis could be reached (37 lung cancer specimens and 37 controls with definite diagnosis). In a total of 36 specimens, no definite diagnosis could be reached (13 lung cancer specimens and 23 nonmalignant samples without definite diagnosis), predominantly due to a low tumor cell content of the specimens. These cases are further classified as “indefinite” or “uncertain diagnosis”. Accordingly, 37 of the 55 patients received a definite diagnosis. DNA methylation values obtained using the HumanMethylation450 BeadChip of a subset of 13 CpG loci were verified by performing 608 bisulfite pyrosequencing reactions in 24 DNA samples isolated from 5 adenocarcinomas, 7 squamous cell carcinoma and 12 controls. The overall Pearson’s correlation coefficient between both techniques was 0.89, which demonstrates high correlation of DNA methylation values determined by independent techniques (Additional file 3: Fig. S2 and Additional file 7: Table S2).

### Aberrant DNA methylation profiles in paired biopsy specimens

**Fig. 1:**
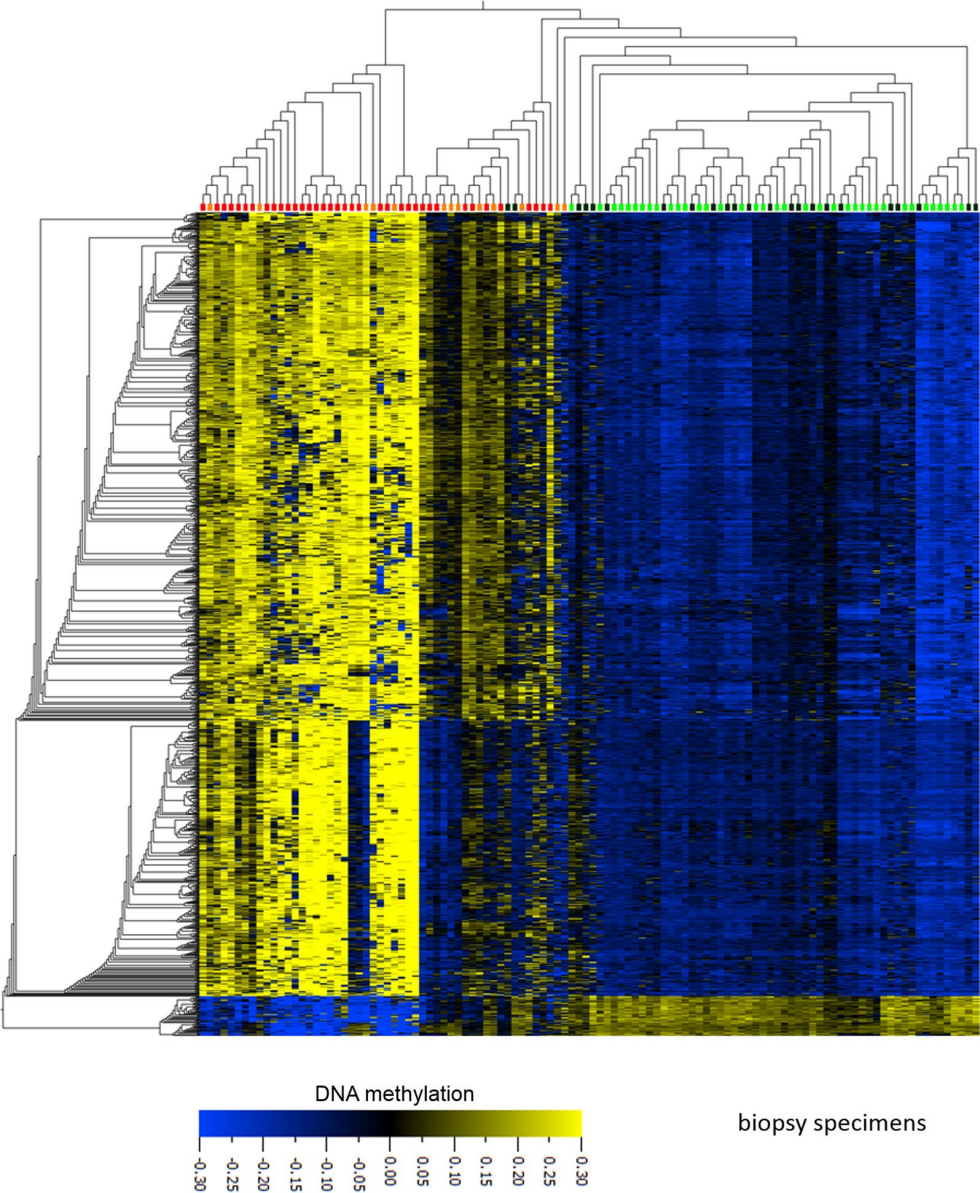
Hierarchical cluster analyses of DNA methylation data. A Wilcoxon paired test statistics (FDR < 1 × 10^–6^, delta. beta > 0.25) applied to DNA methylation data of paired biopsies identified 1303 loci aberrantly methylated in tumor samples (red boxes on top of the heatmap) as compared to the corresponding controls (light green boxes). 34 of the remaining 36 specimens with an uncertain diagnosis (dark green boxes: supposed benign; orange boxes: supposed tumor containing) which were excluded from the statistical test clustered together with their counterparts with certain diagnosis. Two samples with doubtful diagnosis clustered “between” benign and malignant specimens. heatmap: blue: low, yellow: high DNA methylation. For presentation, mean methylation of each locus was normalized to zero (mean = 0)

Differential DNA methylation analysis (DMA) of the paired biopsies with a definite diagnosis available from 37 patients identified 1303 loci (paired Wilcoxon test, FDR < 1 × 10^–6^, delta.beta > 0.25) aberrantly methylated in cancer-cell containing samples as compared to tumor-free samples. A subsequent hierarchical cluster analysis of these 1303 loci including all 110 biopsies clearly separated the specimens with definite infiltration by cancer cells from the corresponding control samples with high specificity and sensitivity (Fig. 1). In the 36 samples labeled as “indefinite diagnosis” by histopathological investigation, the assumption of the pathologist could be verified in the vast majority of samples (32 of 36, 89%). Comparing the pathologist assumption and the outcome of the DNA methylation analysis, the Cohen's kappa coefficient ranged from 0.76 (taking the uncertain diagnosis into account only; Cohens *κ*_uncertain_ = 0.76) to 0.93 (including all 110 samples; Cohens *κ*_overall_ = 0.93). Only two samples with uncertain diagnosis (P05140125T and P10130074T) histologically considered most likely as benign clearly clustered with the tumor-containing samples. Interestingly, patient P10130074T presented clinically as stage 4 lung carcinoma without histological confirmation, whereas specimen P05140125T turned out being a metastasis of a renal carcinoma by a second histological evaluation. Thus, both tumors, which have been histologically misclassified as non-malignant were correctly identified by the epigenetic approach.

In turn, two uncertain diagnosis samples suspicious for being cancerous clustered between benign and malignant samples, both of these were collected from the same patient (P09130061). These samples could not definitely classified as malignant or benign, neither by histology nor by DNA methylation analysis.

A more detailed analysis revealed that of the 1303 differentially methylated loci, only 63 loci were hypomethylated in the cancer-cell containing biopsies as compared to the corresponding controls. These loci were located in 36 known genes encoding for e.g. transcription factors (ZNF423, PEG3, E2F6), chromosome associated proteins (DCTN2, ZNF423, CSPP1), cell adhesion proteins (SIRPB1) and apoptosis controlling factors (CASP8). Strikingly, four genes belong to the olfactory receptor family (OR8H2, OR8K1, OR2M7 and OR4K5). The 63 hypomethylated CpG loci were enriched for localization in the first exon (OR: 6.88, *p* = 2.55 × 10^–06^, chi^2^-test) but depleted for CGIs (OR: 0, *p* = 2.52 × 10^–07^, chi^2^-test) and DNaseI hypersensitive sites (OR: 0.12, *p* = 0.02, chi^2^-test).

In contrast, the hypermethylated loci in tumor containing biopsies mapped to 555 genes. A gene ontology search demonstrated that these genes contributed to known tumor and signaling pathways, i.e. to the TGF-beta signaling pathway (CREB, FBN1, GDF5, PITX2, RGMA, SMAD3, THSD4), the RAS signaling pathway (ABL1, INSR, NTRK1, PIK3CA, PIK3R1, PIK3R2, RAPGEF5, ZAP70), the TNF signaling pathway (DAB2, MAP3K14, MAPK14, PIK3CA, PIK3R1, PIK3R2, RIPK1, TNFAIP3, VCAM1) or apoptosis (CAPN1, DAB2IP, ITPR2, LMNA, MAP3K14, NTRK1, PIK3CA, PIK3R1, PIK3R2, RIPK1, TP53AIP1). In general, hypermethylated loci were found enriched for gene bodies (OR: 1.27, *p* = 2.67 × 10^–4^, chi^2^-test), 5′UTRs (OR: 1.38, *p* = 0.02, chi^2^-test), CGIs (OR: 1.15, *p* = 0.02, chi^2^-test), enhancers (OR: 2.48, *p* = 5.78 × 10^–59^, chi^2^-test) and DNaseI hypersensitive sites (OR: 1.71, *p* = 6.31 × 10^–14^, chi^2^-test).

**Fig. 2:**
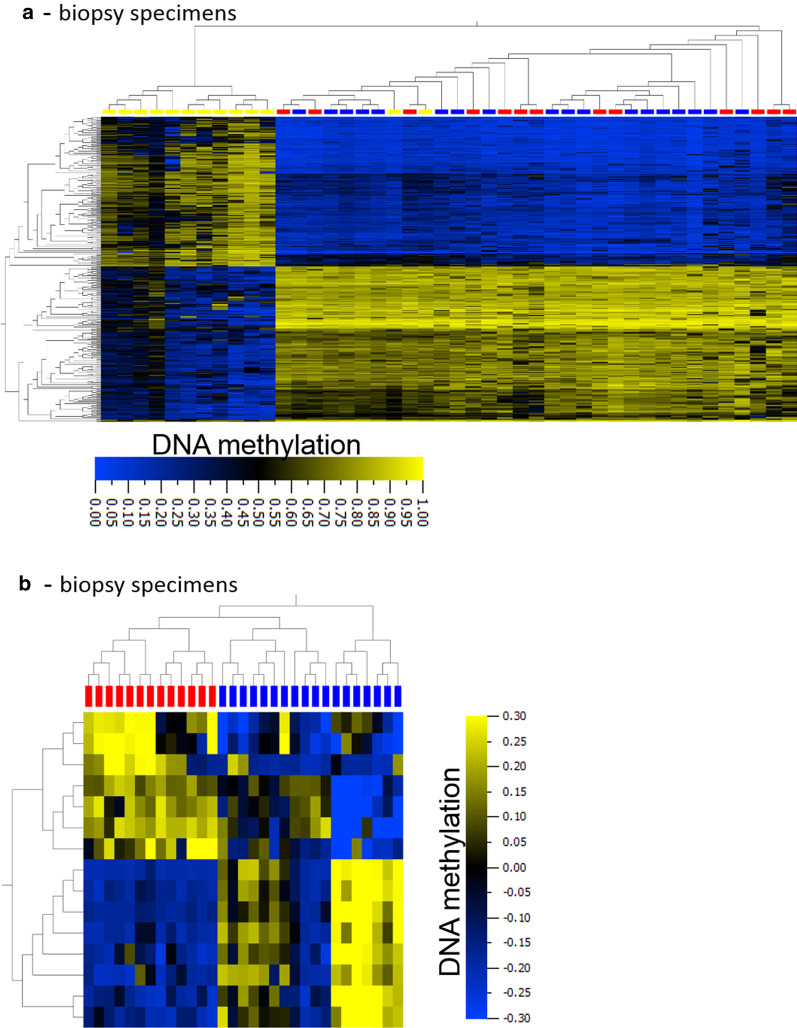
Differential DNA methylation analysis to separate tumor entities. **a** After performing an ANOVA (FDR < 1 × 10^–6^, *σ*/*σ*_max_ > 0.25) a hierarchical cluster analysis of the 300 resulting loci separated SCLC and NSCLC specimens in biopsy samples. Boxes on top of the heatmap: yellow boxes: SCLC, red boxes: AC, blue boxes: SQC. Methylation values are presented without further normalization (avg.beta values). **b** Furthermore, a t-test statistic (FDR < 0.05, *σ*/*σ*_max_ > 0.55) separating AC and SQC in biopsy specimens with certain diagnosis resulted in 15 loci. This set of loci separates both entities in the biopsy cohort in a subsequent hierarchical cluster analysis. Boxes on top of the heatmap: red boxes: AC, blue boxes: SQC. For presentation, mean methylation of each locus was normalized to zero (mean = 0). Heatmap: blue: low, yellow: high DNA methylation

Therapy of lung cancer besides clinical presentation and increasingly mutational findings relies on histopathologic subtyping [7] into SLCL, AC and SQC. To study, whether DNA methylation of biopsy samples from bronchoscopy might add to this subtyping we in the first step investigated differential DNA methylation of lung cancer entities in our cohort by performing an ANOVA analysis. Due to limited sample numbers in the different groups (Additional file 6: Table S1), we focused on SCLC, AC and SQC. Hierarchical cluster analysis of the identified 300 differentially methylated loci (*σ*/*σ*_max_ > 0.25, FDR < 1 × 10^–6^, ANOVA; corresponding to 170 individual genes) resulted in two major branches separating SCLC from NSCLC (AC and SQC) samples with the exception of two samples (Fig. 2a, Additional file 9: Table S3; Cohens *κ* = 0.86). These two have been classified previously as SCLC but belong to the set of specimens with an uncertain histological diagnosis (76701 and P10130072, Additional file 6: Table S1). However, these samples show a DNA methylation pattern different from the one of other SCLC samples but identical to those of the NSCLC specimens, suggesting a misclassification of these biopsies based on the initial histological screening. In a second approach we focused on the clinically most relevant groups of NSCLC, AC (*n* = 13 samples in the biopsy cohort) and SQC (*n* = 18). Applying a t-test statistic to identify loci differentially methylated between these entities revealed 15 CpG loci (q < 0.05, *σ*/*σ*_max_ > 0.55; cg00129651, cg00370229, cg00400827, cg01188578, cg06922248, cg09451235, cg11965913, cg12861034, cg17178900, cg18367631, cg20395967, cg20668644, cg20691436, cg22061831, cg26631039) corresponding to nine individual genes (ARHGEF4, CALML3, GLI2, HADHA, MIR663, PM20D1, PRKAR1B, RAPGEFL1, ZDHHC1). Considering these loci only, a hierarchical cluster analysis of the methylation values separated AC from SQC (Fig. 2b).

### Aberrant DNA methylation profiles in surgical lung cancer specimens and comparison to biopsy specimens

To compare the results obtained from bronchoscopic biopsy samples with high quality specimens usually used in numerous other studies on lung cancer and to further validate our findings, we in addition collected an independent sample cohort of surgical cancer specimens. In all these samples a firm histopathologic diagnosis could be reach (Additional file 6: Table S1). To minimize putative corruption of DNA methylation data in malignant cancer cells due to adjacent non-malignant cells of the tumor microenvironment, tumor cell content was increased by macrodissection (resulting tumor cell content > 80%). In parallel, matched tumor-free control samples were isolated from the same surgically removed tissue.

**Fig. 3:**
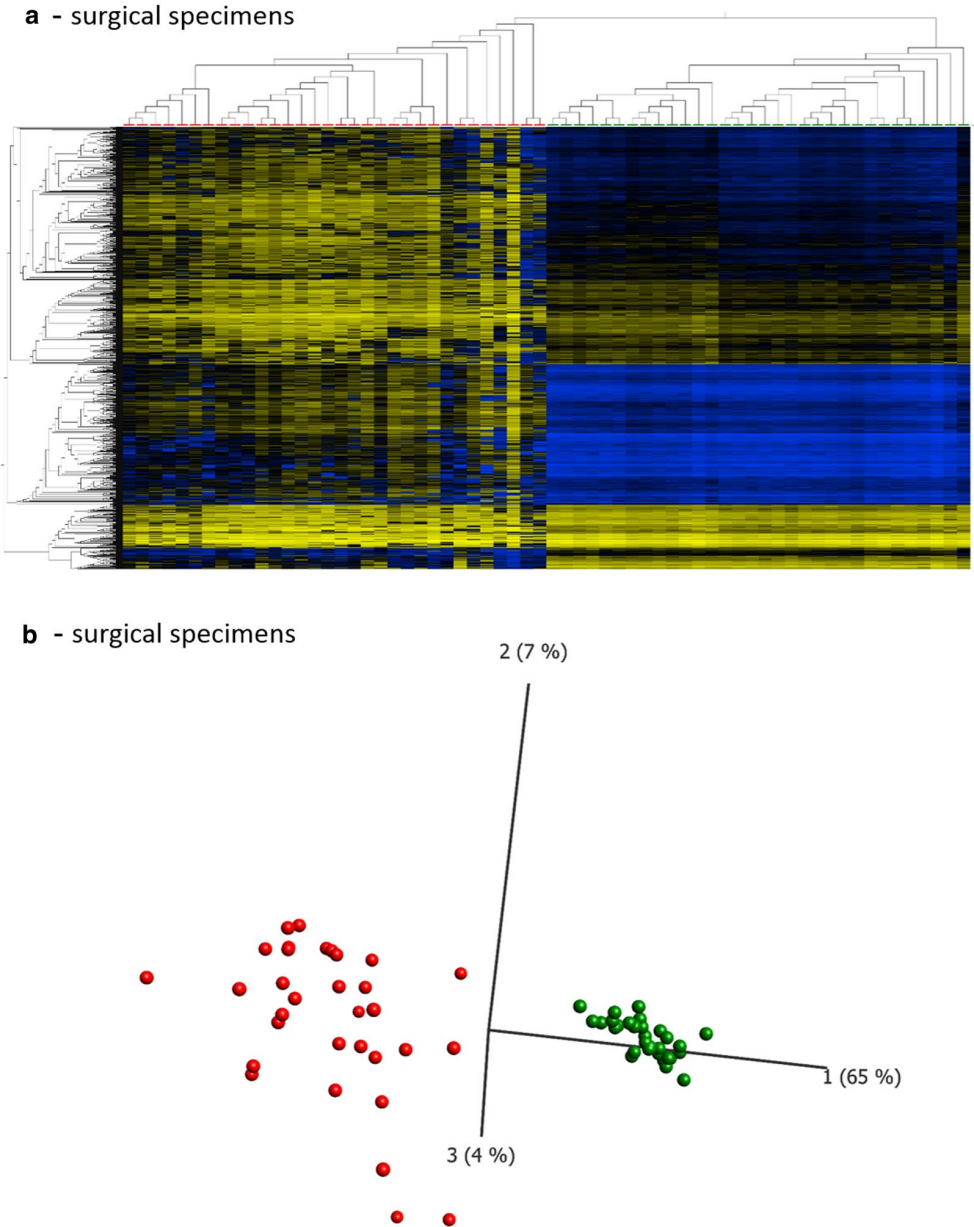
Hierarchical cluster analyses and principal component analysis of surgical specimens. 1303 differentially methylated loci identified by a DMA of paired biopsies were analyzed in the surgical specimens' data set by performing a hierarchical cluster analysis (**a**) or PCA (**b**). Heatmap and PCA: red boxes/spheres: tumor samples, green: control tissue samples; heatmap: yellow: high, blue: low DNA methylation values

**Fig. 4:**
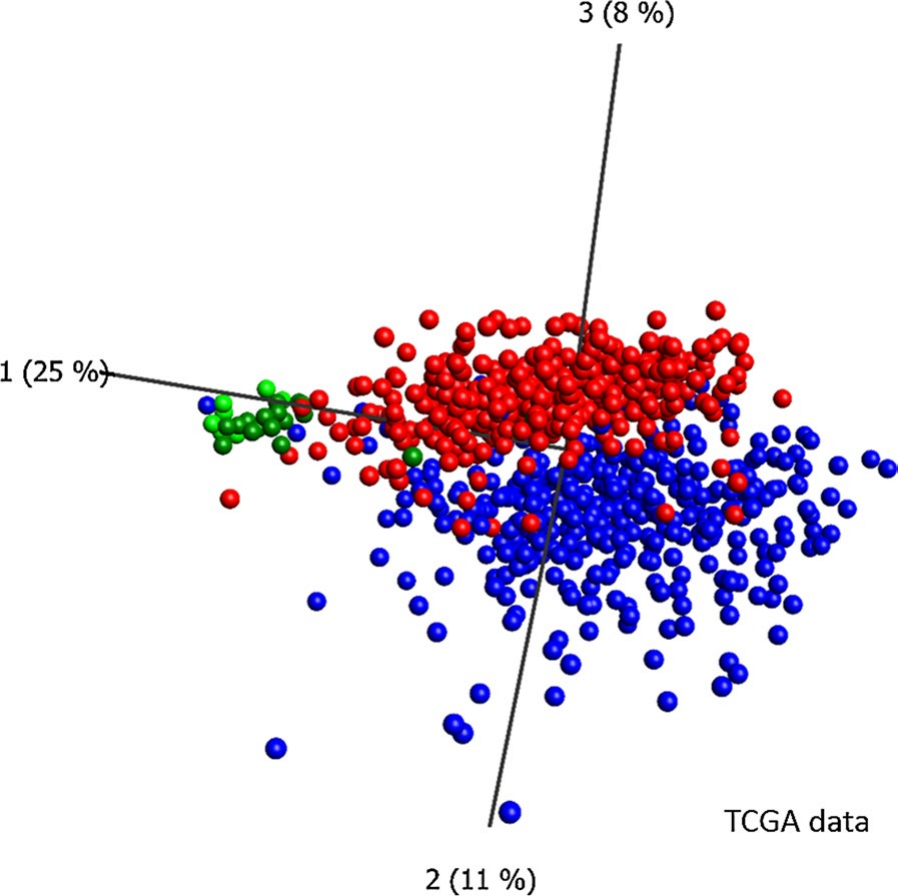
Principal component analysis of TCGA data on lung cancer. TCGA DNA methylation data of 1163 CpG loci present in both, the TCGA data set as well as the set of 1303 differentially loci were subjected to a PCA (unsupervised). Red spheres: AC samples (*n* = 439), blue spheres: SQC samples (*n* = 369), green spheres: control tissue samples (*n* = 26)

In a first approach we investigated how the panel of 1303 CpG loci obtained from the DMA of the paired bronchoscopic biopsy samples described above performed on the independent cohort of surgically resected specimens. Based on the methylation status of these loci all surgical specimens were correctly separated into tumoral and normal by both hierarchical cluster analysis and PCA (Fig. 3a, b). Therefore, the DMA results of the biopsy samples could be fully validated using the independent cohort of surgically obtained primary specimens (*κ* = 1). Additionally, we applied the set of 1303 loci to a DNA methylation data set provided by the TCGA including more than 800 lung cancer samples (439 AC and 369 SQC) and 26 non-malignant controls (see materials). Although this data set contains only information of 1162 of the 1303 loci, both, a subsequently performed PCA (Fig. 4) or hierarchical cluster analysis (Additional file 4: Fig. S3) separated lung cancer and control samples with only a minor number of exceptions. This further confirmed the results of our analysis of surgical specimens. Depending on the selected branch of the cluster analysis, the agreement between histological and epigenetic diagnosis is almost perfect (*κ* = 0.87, Cohens kappa comparing the diagnostic outcome of histological and DNA methylation based analyses).

Further analyses in particular of the data set obtained from the surgical specimens as well as a comparison with the results obtained from bronchoscopic biopsies is provided in the Additional file 1: Supplement.

## Discussion

This study aimed at analyzing CpG loci aberrantly methylated in paired biopsy specimens collected during bronchoscopy of lung cancer patients, compared to highly pure, clinically and histologically well characterized surgical specimens. DNA methylation data were collected by array analysis. Reliability of array data was ensured by bisulfite pyrosequencing of arbitrary selected loci.

In a first approach, a classical statistical analysis to identify aberrantly methylated CpG loci in bronchoscopy specimens of lung cancer patients and corresponding controls revealed 1303 loci (FDR < 1 × 10^–6^, delta.beta > 0.25). This panel of loci separated reliably benign from malignant specimens in the biopsy cohort (Cohens *κ*_overall_ = 0.93, even if taking the misclassified cases into account), the cohort of surgical specimens (*κ* = 1) as well as in the TCGA sub-cohort (*κ* = 0.87, although the TCGA sub-cohort available contains only information of 1162 of the 1303 loci). This demonstrates and underlines the capability of DNA methylation analysis for diagnosing complex clinical lung cancer cases. Interestingly, also several challenging specimens which have been misclassified by the pathologist have been correctly identified by the epigenetic approach.

Only a minority of 63 of the 1303 aberrantly methylated loci were hypomethylated in cancer samples as compared to controls. The 36 genes corresponding to these loci included several transcription factors with a known impact on carcinogenesis (i.e. ZNF423, PEG3 and E2F6), chromosome associated proteins like CSpp1 or several members of the olfactory receptor family. Low expression of the transcription factor ZNF423 induces growth of tumor cells and correlates with a poor clinical outcome in neuroblastoma patients [12, 13]. Furthermore, by performing a motif analysis of tumor-specific methylated regions in SCLC Kalari et al. identified an enrichment of binding sites for several transcription factors including ZNF423 supposing a functional role of this factor in lung cancer [14]. PEG3 is supposed to affect cell proliferation and apoptosis mediated by p53. This factor, which is expressed only from the paternally inherited allele acts as tumor suppressor in ovarian cancers as well as in gliomas [15, 16]. Also E2F6 acts as transcription regulator. Overexpression of E2F6 in combination with deregulation of distinct other factors has been suggested as biomarker for AC and SQC in peripheral blood [17]. The chromosome associated protein CSPP1 regulates cell-cycle progression, spindle organization [18] and regulates cytokinesis. However, its impact in lung cancer remains yet unclear. Interestingly, four of the 36 hypomethylated genes belonged to the olfactory receptor family (OR8H2, OR8K1, OR2M7 and OR4K5). These are members of the family of G-protein-coupled receptors. While their specific function in many tissues, including lung tissue, is still speculative, they have been shown to be involved in numerous cellular processes including cell–cell recognition, migration, proliferation and apoptosis [19]. Nevertheless, other members of the olfactory receptor family have already been shown to play a role in cancer including lung carcinomas. OR51E1 for example has been suggested as target for diagnosis in somatostatin receptor-negative lung carcinoids [20], whereas OR3A4 promotes cisplatin resistance of non-small cell lung cancer [21].

The vast majority of loci were hypermethylated as compared to controls. In particular CGIs, enhancers and 5′UTRs were affected by hypermethylation, which is in line with other reports [8]. Interestingly, we found several relevant pathways (i.e. the TGFbeta, RAS, TNF or apoptosis signaling pathways) in tumors affected by aberrantly methylated genes, which might propose a functional impact of the altered DNA methylation in these cases. Deregulation of TGFbeta signaling has been correlated with EMT transition, cell migration and invasion in lung cancer [9] whereas TNF signaling is being expected playing a major role in inflammation-induced cancer and disturbances in the RAS signaling in lung cancer are long known [10]. Interestingly, TNF has even been suggested as biomarker for NSCLC [11]. Numerous genes found aberrantly methylated in this study contribute to these signaling pathways and are well known to play a role in lung cancer as well as in carcinogenesis in general. For example SMAD3 is a member of the TGFbeta signaling pathway. It acts as tumor suppressor and regulates cell proliferation. An effect of SMAD3 in smoking induced resistance to chemotherapy as well as the attenuation of the tumor suppression function of TGFbeta in lung cancer has been shown [22, 23]. Like SMAD3, also PITX2 is a member of the TGFbeta signaling pathway. It belongs to the bicoid class of homeodomain proteins and is involved in the development of several organs. PITX2 has been shown to enhance progression of lung adenocarcinoma [24]. Furthermore, the DNA methylation status of PITX2 and SHOX2 has been suggested to predict the outcome in patients with NSCLC [25]. ABL1 is a protooncogene that encodes a protein tyrosine kinase. ABL1 contributes to the RAS signaling pathway and is involved in a variety of cellular processes with an impact on carcinogenesis, including cell differentiation, cell adhesion and division. Somatically mutated ABL1 has been suggested to be essential for the survival of NSCLC cells [26]. Furthermore, ABL1 can promote metastasis of lung cancer cells carrying also EGFR or KRAS mutations [27]. Another member of the RAS signaling pathway, PIK3CA encodes for the catalytic subunit of the Phosphatidylinositol 3-kinase. It has been described to be oncogenic. After reviewing several publications that investigated the impact of PIK3CA on lung cancer, Wang et al. concluded that PIK3CA mutation may not only affect lymph node metastasis but might also serve as prognostic factor in NSCLC. Additionally, smoking may be correlated with an increase in PIK3CA expression [28]. Besides the catalytic subunit we also found the phosphoinositide-3-kinase regulatory subunits 1 and 2 to be aberrantly methylated in tumor specimens of our biopsy cohort. Both subunits have been shown to play a role in malignancies including lung cancer [29, 30]. Consequently, the results of this study are in line with current reports in the literature.

A subsequently performed ANOVA including malignant specimens only revealed 300 loci separating SCLC from AC and SQC, corresponding to 170 genes (*σ*/*σ*_max_ > 0.25, FDR < 1 × 10^–6^, Cohens *κ* = 0.86). According to the KEGG Mapper nine of these genes contribute to pathways in cancer, i.e. AKT3, APPL1, AXIN1, CTBP2, GNG4, NFE2L2, NOTCH1, NOTCH3 and PDGFA. AKT3 increases migration and metastasis in cancer cells [31], while a knockdown of this gene induces mitochondrial dysfunction [32]. APPL1 regulates cell proliferation and migration in malignant cells [33] and AXIN1 acts as a negative regulator of the WNT1 pathway. Hypermethylation of this gene with clinical significance in lung cancer has been already reported before [34]. CTBP2 affects the WNT in NSCLC cells [35] and furthermore activates TGF-beta signaling [36], while NFE2L2 encodes a transcription factor which has been recurrently reported being altered in NSCLC [37]. Finally, members of the NOTCH receptor family are important regulators of cell interactions. Alterations have been reported in multiple cancers, including lung cancer but also in other lung diseases [38]. Besides the KEGG cancer pathway, also genes contributing to other signaling pathways like the notch signaling pathway (*p* < 0.0001, Bayes Factor: 6) were affected.

In summary, our results indicate that DNA methylation analysis might be a promising supplementary tool to characterize biopsy specimens that do not allow diagnosis based on pathological findings alone.

## Conclusions

Lung tissue biopsies collected during bronchoscopy of limited quality and low or undefined tumor cell content can pose challenges in establishing a definite diagnosis. In our hands DNA methylation proofed of high value not only to detect tumor cell DNA present in the sample but also to determine the tumor entity.

Furthermore, our data indicate that panels of CpG loci built on high quality sample material might be less favorable when analyzing biopsy samples with low tumor cell content or of low quality (see Additional file 1). This might make an impact on future studies building or applying diagnostic DNA methylation panels.

## Methods

### Tissue specimens

55 paired biopsy specimens of lung cancer patients from suspicious (tumor) lesions as well as corresponding tumor free control tissues (mostly from the contralateral bronchi) were collected during bronchoscopy. All samples underwent extensive histological examination by trained pathologists for diagnosis as well as for estimating tumor cell content. In this study, we intentionally included also numerous paired biopsies (*n* = 18) with at least one sample showing an uncertain histological pattern resulting into a difficult and (without further investigations and clinical information) uncertain diagnosis. In these cases the pathologists made a decision based on their experience and only the material available.

As part of a second, independent and unmatched sample cohort, surgically resected lung tumor tissue specimens as well as matched tumor-free control tissue samples of 15 patients suffering from adenocarcinomas (AC) and 18 patients with squamous cell carcinomas (SQC) who underwent surgery with curative intent were collected. Tumor specimens have been macrodissected to enrich tumor cell content. Tumor cell content > 80% has been confirmed by trained pathologists. The results from all histological analyses are presented in Additional file 6: Table S1. Ethical permission was obtained from the University of Lübeck through the Biomaterialbank North (Ref. 12-220 and Ref. 12-238). Subsequent DNA isolation and bisulfite conversion was performed as detailed in [39].

### Methylome analysis

After extraction of the DNA, methylome data was generated as previously reported using the HumanMethylation450K BeadChip (Illumina, Inc., San Diego, CA, USA) according to the manufacturer’s instruction. This chip allows the parallel methylation analysis of 485,577 loci. Data was stored in GEO (accession number GSE158075). Raw data analysis was performed using the GenomeStudio software package (Illumina, Inc.). CpG loci located on gonosomes as well as CpG loci with detection. *p*-values > 0.01 were excluded from further analyses. Surgical and paired biopsy specimens were normalized and analyzed separately. After exporting the resulting data, Omics Explorer 3.2 (Qlucore, Lund, Sweden) was used for subsequent cluster analyses, PCA, machine learning approaches and data presentation. The Gorilla tool [40], GATHER and KEGG tools [41] were used for gene ontology analyses, db-string for interaction analyses [42]. PERL (ver. 5), Julia (ver. 0.6) as well as R (ver. 3.4.3) were used to perform statistical analysis as detailed in the figure legends and the text and for generating figures. While in particular the control samples shared a similar DNA methylation pattern, methylation was more heterogeneous in malignant samples (most obvious in surgical specimens with high tumor cell content, Additional file 5: Fig. S4). For validation purposes we used *The Cancer Genome Atlas* (TCGA) data publically accessible from BROAD institute on 439 AC and 369 SQC [43].

### Bisulfite pyrosequencing (BSPS)

Bisulfite pyrosequencing was performed for data validation as detailed in [44]. A list of PCR primers used and conditions applied for PCR amplification are provided in Additional file 9: Table S4.

## Supplementary Information

**Additional file 1. Supplement.** This file contains further analyses in particular of the data set obtained from the surgical specimens as well as the comparison with the results obtained from bronchoscopic biopsies.

**Additional file 2. Fig. S1**: Cohorts and samples included into the study. (**A**) The first cohort consisted of 110 paired bronchoscopic biopsy specimens from 55 patients. From each patient two biopsies have been collected, one from the suspicious tumor lesion and another one from the contralateral bronchus. Based on the histological examination by trained pathologists 37 patients (corresponding to 74 individual biopsies) received a definite diagnosis of lung cancer (15 AC, 19 SQC, 3 other lung cancer entities). From those also 37 control samples were included. The remaining 18 patients (corresponding to 36 individual biopsies) did not receive a final diagnosis, 13 biopsies were classified as "tumor suspicious", 23 as "probably non-malignant". (**B**) The second sample cohort consisted of 32 surgically removed lung cancer specimens. From each specimen tumor cells were enriched by macrodissection (tumor cell content >80%), resulting in 32 tumor samples (14 AC and 18 SQC). Non-malignant lung tissue samples were collected from the periphery of the surgically specimens (32 non-malignant control samples). The minimum distance between the sampling sites of tumor specimen and control specimen was 1cm. (**C**) For in silico analyses a DNA methylation data set provided to the public by the TCGA consortium has been used (439 AC-, 369 SQC- samples and 26 control specimens)

**Additional file 3. Fig. S2**: Validation of DNA methylation values collected by HumanMethylation450 BeadChip (HM450 BC) using bisulfite sequencing (BSPS). For verifying results obtained by array analysis, 13 CpG loci were selected and BSPS assays were designed. These loci were subsequently analyzed in both malignant and benign samples of surgical and biopsy specimens. Overall 608 BSPS reactions were performed for HM450 BC data verification. Afterwards, BSPS data was correlated with data obtained from HM450 BC analysis by determining the Pearson’s correlation coefficient. The results of the overall analysis as well as the analysis of four specimens’ subgroups are shown. Additional BSPS assays succeeded to validate loci differentially methylated between SQC and AC as determined by HM450 BC analysis (data not shown). Data sets from the following CpG loci were included: cg04415798, cg23322933, cg18103859, cg05877497 (24 surgical specimens and 24 biopsy samples: 12 tumors, 12 controls each); cg22620090, cg06809252 (24 surgical specimens: 12 tumors, 12 controls and 22 biopsy samples: 11 tumors, 11 controls), cg13588800, cg20052718, cg17839237, cg24446548 (34 surgical specimens: 17 tumors, 17 controls and 24 biopsy samples: 12 tumors, 12 controls), cg02391713 (24 surgical specimens: 12 tumors, 12 controls), cg00240432 and cg14782672 (34 surgical specimens: 17 tumors, 17 controls).

**Additional file 4. Fig. S3**: Hierarchical cluster analyses of TCGA data on lung cancer. 1162 of the 1303 differentially methylated loci identified in the DMA of paired biopsies of which methylation data are available in the TCGA data set, were analyzed in the TCGA data set by performing a hierarchical cluster analysis. heatmap: red boxes: AC samples, blue boxes: SQC samples, green boxes: control tissue samples; heatmap: yellow: high, blue: low DNA methylation values.

**Additional file 5. Fig. S4**: Correlation matrix of DNA methylation data collected from 40 surgical tumor specimens and their corresponding control using the HumanMethylation450 BeadChip. DNA methylation data of all surgical specimens included into this study were subjected to correlation analysis by first calculating Pearson’s correlation coefficient for each combination of sample pairs and subsequently building a correlation matrix. Red bar below and right of the matrix: tumor samples, green bar: controls. Matrix and bar below of the matrix: blue: low correlation coefficient, yellow high correlation coefficient. While control samples are characterized by high homogeneity of their methylation values, the tumor methylome is highly heterogeneous.

**Additional file 6. Table S1**: Table of surgical specimens (1st sheet) and paired biopsies (2nd sheet) included into this study. Additional clinical information known to the authors is included.

**Additional file 7. Table S2**: Results of the analysis of 608 individual BSPS reactions to validate methylation values collected by HM450 BC and comparison to array data. See also Additional file 3: Fig. S2.

**Additional file 8. Table S3**: ANOVA to identify loci differentially methylated between AC, SQC and SCLC.

**Additional file 9. Table S4**: Table of primers and primer sequences for performing bisulfite pyrosequencing (BSPS). The annealing temperature [°C] applied is shown (Tm).

## Data Availability

The datasets generated and/or analyzed during the current study are available in the gene expression omnibus (GEO) repository, GSE75008 and GSE158075.

## Abbreviations

CGI: CpG Island
PCA: Principal component analysis
PCR: Polymerase chain reaction
TCGA: The cancer genome atlas program
ICGC: International cancer genome consortium
OR: Odds ratio
5′UTR: 5′-Untranslated region
AC: Adenocarcinoma
SQC: Squamous cell carcinoma
SCLC: Small cell lung carcinoma
NSCLC: Non-small cell lung carcinoma
FDR: False discovery rate
GO: Gene ontology
DMA: Differential DNA methylation analysis
