# Supplementary figures and images for: DNA methylation profiles of bronchoscopic biopsies for the diagnosis of lung cancer
Source: Clin Epigenetics. 2021 Feb 17;13:38. doi: 10.1186/s13148-021-01024-6 (PMC7890863; doi:10.1186/s13148-021-01024-6)

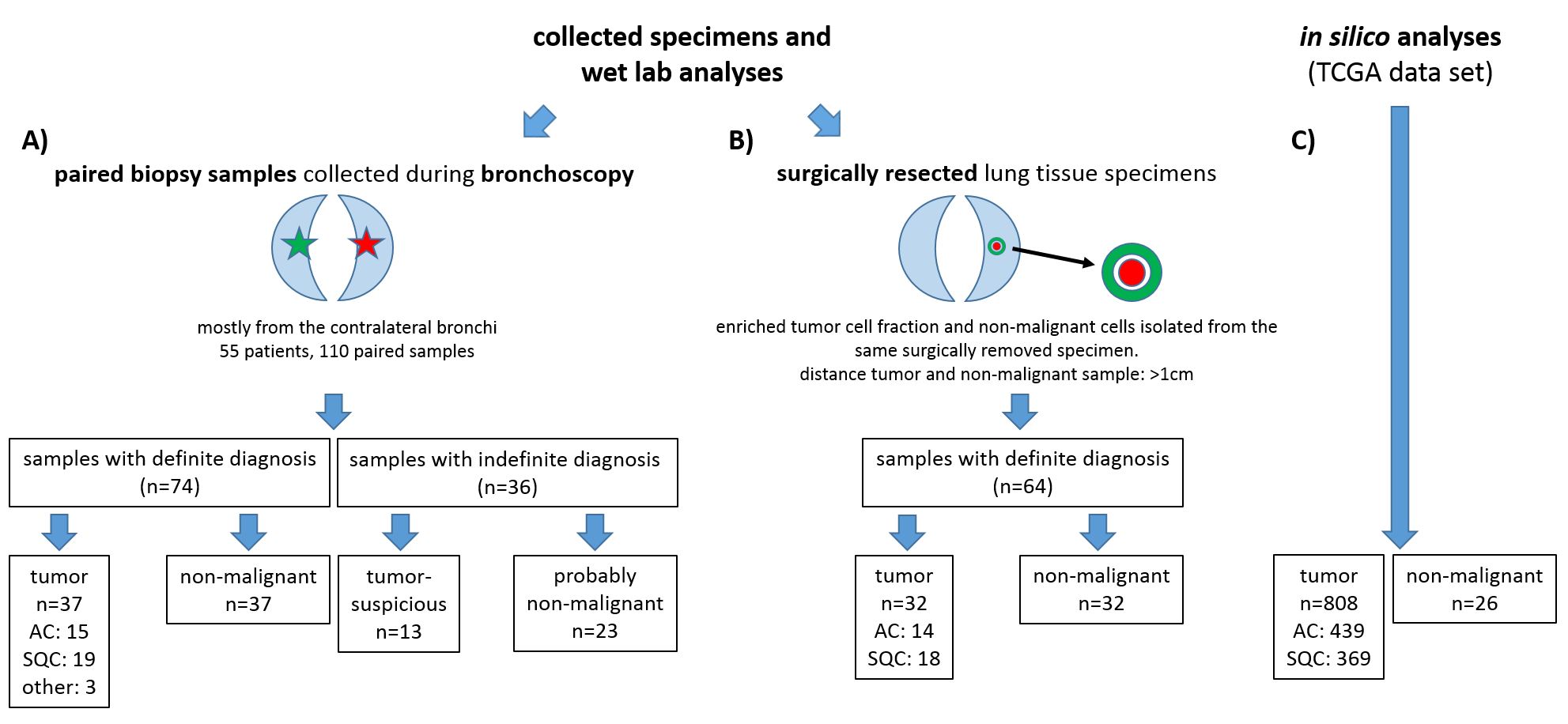

Supplement: Supplementary file 1 — Additional file 1. Supplement. This file contains further analyses in particular of the data set obtained from the surgical specimens as well as the comparison with the results obtained from bronchoscopic biopsies. [file 13148_2021_1024_MOESM1_ESM.png]

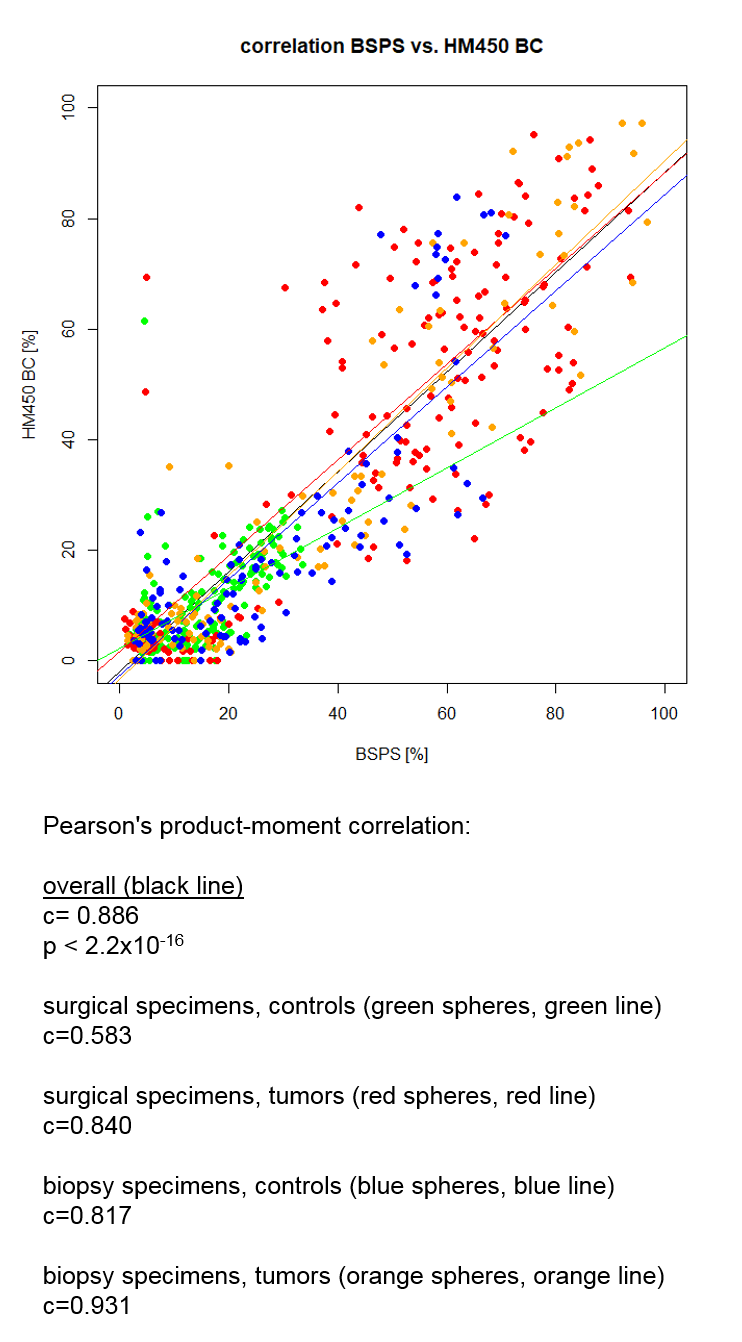

Supplement: Supplementary file 2 — Additional file 2. Fig. S1: Cohorts and samples included into the study. (A) The first cohort consisted of 110 paired bronchoscopic biopsy specimens from 55 patients. From each patient two biopsies have been collected, one from the suspicious tumor lesion and another one from the contralateral bronchus. Based on the histological examination by trained pathologists 37 patients (corresponding to 74 individual biopsies) received a definite diagnosis of lung cancer (15 AC, 19 SQC, 3 other lung cancer entities). From those also 37 control samples were included. The remaining 18 patients (corresponding to 36 individual biopsies) did not receive a final diagnosis, 13 biopsies were classified as "tumor suspicious", 23 as "probably non-malignant". (B) The second sample cohort consisted of 32 surgically removed lung cancer specimens. From each specimen tumor cells were enriched by macrodissection (tumor cell content >80%), resulting in 32 tumor samples (14 AC and 18 SQC). Non-malignant lung tissue samples were collected from the periphery of the surgically specimens (32 non-malignant control samples). The minimum distance between the sampling sites of tumor specimen and control specimen was 1cm. (C) For in silico analyses a DNA methylation data set provided to the public by the TCGA consortium has been used (439 AC-, 369 SQC- samples and 26 control specimens) [file 13148_2021_1024_MOESM2_ESM.png]

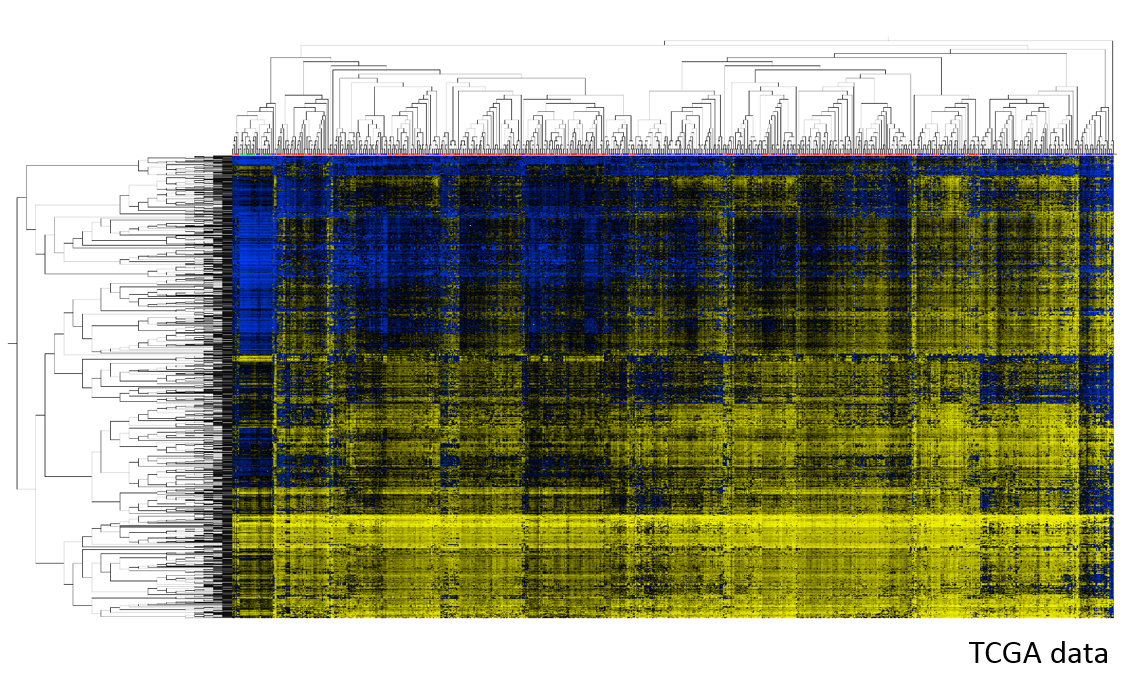

Supplement: Supplementary file 3 — Additional file 3. Fig. S2: Validation of DNA methylation values collected by HumanMethylation450 BeadChip (HM450 BC) using bisulfite sequencing (BSPS). For verifying results obtained by array analysis, 13 CpG loci were selected and BSPS assays were designed. These loci were subsequently analyzed in both malignant and benign samples of surgical and biopsy specimens. Overall 608 BSPS reactions were performed for HM450 BC data verification. Afterwards, BSPS data was correlated with data obtained from HM450 BC analysis by determining the Pearson’s correlation coefficient. The results of the overall analysis as well as the analysis of four specimens’ subgroups are shown. Additional BSPS assays succeeded to validate loci differentially methylated between SQC and AC as determined by HM450 BC analysis (data not shown). Data sets from the following CpG loci were included: cg04415798, cg23322933, cg18103859, cg05877497 (24 surgical specimens and 24 biopsy samples: 12 tumors, 12 controls each); cg22620090, cg06809252 (24 surgical specimens: 12 tumors, 12 controls and 22 biopsy samples: 11 tumors, 11 controls), cg13588800, cg20052718, cg17839237, cg24446548 (34 surgical specimens: 17 tumors, 17 controls and 24 biopsy samples: 12 tumors, 12 controls), cg02391713 (24 surgical specimens: 12 tumors, 12 controls), cg00240432 and cg14782672 (34 surgical specimens: 17 tumors, 17 controls). [file 13148_2021_1024_MOESM3_ESM.png]

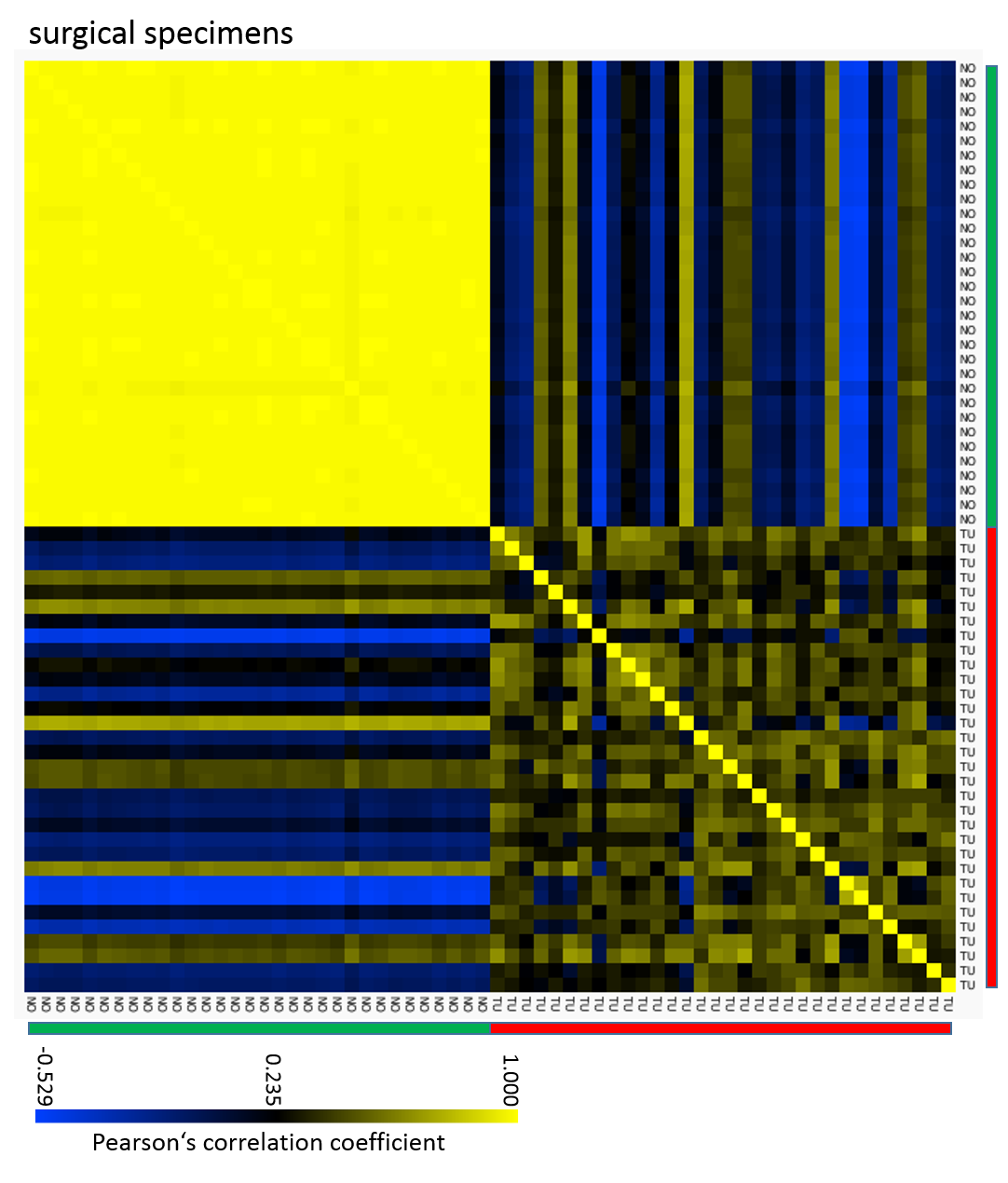

Supplement: Supplementary file 4 — Additional file 4. Fig. S3: Hierarchical cluster analyses of TCGA data on lung cancer. 1162 of the 1303 differentially methylated loci identified in the DMA of paired biopsies of which methylation data are available in the TCGA data set, were analyzed in the TCGA data set by performing a hierarchical cluster analysis. heatmap: red boxes: AC samples, blue boxes: SQC samples, green boxes: control tissue samples; heatmap: yellow: high, blue: low DNA methylation values. [file 13148_2021_1024_MOESM4_ESM.png]
