# Supplementary material for: DNA methylation profiles of bronchoscopic biopsies for the diagnosis of lung cancer
Source: Clin Epigenetics. 2021 Feb 17;13:38. doi: 10.1186/s13148-021-01024-6 (PMC7890863; doi:10.1186/s13148-021-01024-6)
Supplement: Supplementary file 8 — Additional file 8. Table S3: ANOVA to identify loci differentially methylated between AC, SQC and SCLC. [file 13148_2021_1024_MOESM8_ESM.pdf]

Supplementary table 8: Bisulfite pyrosequencing (BSPS)

	CpG	Primer Name	Sequenz (5'→3')	
1	cg04415798	PAX9 Fw	TTGAGTTTAGAGTGAGTAGTGTAATTAG	Biotin
		PAX9 Rv	CCAACAAAAAAAAATAATACTAAAACCTTC	
		PAX9 Seq	AAAAAAAAATAATACTAAAACCTTC	
2	cg23322933	cg233 Fw	TGGGTTTGGGAGTTGGTT	Biotin
		cg233 Rv	CAACTCCAAAAACAAAAAACCTAATAAC	
		cg233 Seq	CTAAACTTCCTAATACAACTCAACT	
3	cg18103859	cg18 Fw	TGTTTTAGTTTAGAGAATAGGTTTAAGA	Biotin
		cg18 Rv	ACTAAAAACCAAACTTCCTAAATTCTCA	
		cg18 Seq	AAACCAAAAAATTTAACTCC	
4	cg05877497	Meis 1 Fw	AGGTTGGTTGTAAATGTTTTTGT	
		Meis 1 Rv	CAAAATTTAAACACACATACTAACAAACC	Biotin
		Meis 1 Seq	GGGATAAGATTTAGGGGAAATA	
5	cg02391713	cg023 Fw	TATTTGTTTTTATGTAGTAGGAATGTTAAGGAAAG	
		cg023 Rv	ATTTTCCAAATCTCTCTCTACCCCTAAC	Biotin
		cg023 Seq	GGAAGGAATTAGGATAGT	
6	cg22620090	cg226 Fw	GGAATGTTAAGAAAGTTATAGTTAAGTGGTTTAT	
		cg226 Rv	AAATCTCTCTCTACCCCTAACA	Biotin
		cg226 Seq	AGGAAGGAATTAGGATAGT	
7	cg06809252	ALX3 Fw	TATGGTAGAGTAGATAAGGTGGGTG	
		ALX3 Rv	CCTAAAAACAAAAACCTAACAAACTAATC	Biotin
		ALX3 Seq	AGATAAGGTGGGTGTA	
8	cg13588800	Twist_1 Fw	AGAATGTAGAGGTGTGAGGATG	Biotin
		Twist_1 Rv	CCTTAAACTAAAATCCTTCCAAATTATTCA	
		Twist_1 Seq	AATAAAAACAATACTCACTAAT	
9	cg20052718	Twist_3 Fw	GGTTGTTGTAGGTTTGGTTTTTTT	
		Twist_3 Rv	CCCCCCTCTTCTCCTCTACCC	Biotin

		Twist_3 Seq	TGGTTTTTTTTAGTTGTTGTTTAG	
10	cg17839237	Twist_4 Fw	GGGAGGGGAAGGTGTGGAT	
		Twist_4 Rv	AAACCTCCAAATCTACAACTCT	Biotin
		Twist _4 Seq	TGGATGGTTTAGAGGTTTAAAAAG	
11	cg24446548	Twist_5_Fw	GTGTGGATGGTTTAGAGGTTTAAAAAGAAA	Biotin
		Twist_5_Rv	TAACACTACTACCCCCAAACTTTC	
		Twist_5_Seq	ACACTACTACCCCCAAAC	
12	cg00240432	Twist_6_Fw	GTGGATGGTTTAGAGGTTTAAAAAG	Biotin
		Twist_6_Rv	ACTAAAAAAACCAAAACTTTCCTATAAAACTTC	
		Twist_6_Seq	CAAACCAATAACACTACTAC	
13	cg14782672	Twist_8_Fw	AGGGATTTTTAGAAGTTTTATAGGAAAG	Biotin
		Twist_8_Rv	ACCCACTATATAAAAACTATTACCATTACT	
		Twist_8_Seq	ATTACCATTACTACTATC	
