# Supplementary material for: DNA methylation profiles of bronchoscopic biopsies for the diagnosis of lung cancer
Source: Clin Epigenetics. 2021 Feb 17;13:38. doi: 10.1186/s13148-021-01024-6 (PMC7890863; doi:10.1186/s13148-021-01024-6)
Supplement: Supplementary file 9 — Additional file 9. Table S4: Table of primers and primer sequences for performing bisulfite pyrosequencing (BSPS). The annealing temperature [°C] applied is shown (Tm). [file 13148_2021_1024_MOESM9_ESM.docx]

**Supplementary Information**

Classifier built to separate malignant from non-malignant bronchoscopic biopsy samples.

Since parallel DNA methylation analysis of 1,303 loci in routine praxis would be costly and time consuming we wondered whether a classifier based on the 1,303 loci could be built using the 37 paired biopsies with firm diagnosis. We applied three independent algorithms for building classifiers (K nearest neighbor, KNN, support vector machine, SVM and random tree, RDT). All algorithms resulted in the same set of 10 CpG loci (cg01634146, NFIX; cg01812400, XRCC3; cg02857325, MTHFSD; cg05966228, CTCF; cg09159452, IQCE; cg09957864, GPT; cg13866190, FLJ14107/BIN3; cg17823004, RAI14; cg18025275, FLJ14107/BIN3; cg22630169, NFIX). The resulting classifier not only separated all biopsy specimens with definite diagnosis expectedly (accuracy=1) but also correctly separated more sophisticated biopsy samples with low or no tumor cell content and indefinite diagnosis (supplementary figure 5A), except the four samples found misclassified by the statistic approach as described in the main text and one proposed benign sample (P07130044T) which was classified as malignant in our approach (κ_uncertain_=0.70, κ_overall_=0.91). Interestingly, the latter has been classified as likely malignant in an independent second histological survey, supporting the value of epigenetic analysis in the diagnosis.

Furthermore, when applying the classifier built on biopsy data to the surgical sample cohort, all non-malignant samples clustered together to one main branch in a hierarchical cluster analysis (supplementary figure 5B).

The 10 loci of the classifier corresponded to 8 genes. 2 of these genes (NFIX and FLJ14107/BIN3) were represented by 2 loci. 4 of 10 loci are located in known enhancers (RAI14, BIN3, CTCF and MTHFSD). Most of these genes were already described in the context of cancer or lung cancer in particular: members of the nuclear factor 1 family are key regulators of mammalian development, which also act as epigenetic regulators within tumors [1]. In particular NFIX was revealed as master regulator of 17 genes involved in migration and invasion pathways [2]. In the study by Rahman et al., silencing of NFIX resulted in reduced expression of numerous genes as well as cancer-related processes like proliferation, migration and invasion. Moreover, hypomethylation of a CpG site in NFIX was found highly associated with pack-years of cigarette smoking [3]. However, this site was different from the ones identified in our study. High expression of RAI14 was recently reported in numerous lung cancer patients and in the lung cancer cell line A549. It was associated with a super enhancer element and was suggested as a new biomarker for lung AC [4]. Furthermore, RAI14 was identified as a member of a set of eight genes showing significant gene-drug correlation with classical chemotherapeutics [5]. XRCC3 encodes a member of the RecA/Rad51-related protein family. It is involved in homologous recombination and DNA damage repair. Genetic variants and polymorphisms are suggested to be associated with lung cancer risk and therapy response [6, 7, 8]. Besides other genes, methylation of XRCC3 was associated with expression of immune checkpoints in several tumors, including lung cancer. The authors of this study suggested that the methylation status of XRCC3 could contribute to a new predictive biomarker for immune checkpoint inhibition [9]. While to our knowledge not yet described in lung cancer, polymorphisms of MTHFSD were described modifying the risk of pancreatic cancer in smoking individuals [10]. Another component of our classifier, CTCF, is a zinc-finger protein. Depending on the binding element on the DNA, it can either act as transcriptional activator or insulator. Upregulation of CTCF was associated with shorter disease-free survival of patients in primary hepatocellular carcinomas [11]. Variants of IQCE have been associated with the risk for polydactyly and autism. To our knowledge, a contribution of IQCE to develop cancer remains yet unknown. Expression of GPT in cancer, including lung cancer, has been investigated already decades ago [12]. However, increased levels of GPT are not restricted to malignancies. BIN3 intronic transcript 1 is a member of a set of 10 genes which expression predicts the relapse in patients with breast cancer treated with tamoxifen [13]. Moreover, deletions of FLJ14107/BIN3 increased susceptibility to lymphoma during aging [14]. Nevertheless, the individual impact of these aberrantly methylated CpG loci in lung cancer has to be addressed by future studies.

Analysis of loci differentially methylated between tumor sampes and controls in surgical specimens and comparision loci identified in bronchoscopic biopsies.

In a further approach we first analyzed the DNA methylation data of the surgical specimens and subsequently compared the results with those of the biopsies. Interestingly, already an unsupervised principal component analysis of the complete data set of the surgical specimens separated not only benign from malignant, but also most of the AC from SQC specimens (supplementary figure 6), indicating the capability of epigenetic data for tumor analysis. Subsequently, by applying a Wilcoxon paired test, 10,098 CpG loci differentially methylated between malignant and benign surgical samples have been identified (FDR<1x10^-6^, delta.beta>0.25). These corresponded to 2,537 individual genes. Hierarchical cluster analysis of these loci resulted in separation of all tumor and corresponding normal samples (supplementary figure 7A). The affected genes contribute to numerous biological processes, including regulation, development, cell proliferation and growth (supplementary table 5).

Nevertheless, in a hierarchical cluster analysis of these 10,098 loci in the cohort of biopsy specimens with definite diagnosis (n=37 pairs), four lung cancer samples clustered with control specimens while two benign specimens clustered with tumor samples (κ=0.83, supplementary figure 8).

To identify loci commonly aberrantly methylated in both, surgically resected and bronchoscopic biopsy specimens, we intersected the differentially methylated 10,098 loci between the malignant and benign surgical specimens and the 1,303 loci differentiating tumor biopsies and corresponding controls. Interestingly, the DNA methylation values of the resulting 502 loci (corresponding to 192 genes) not only clearly separated malignant and benign surgical specimens in a subsequent hierarchical cluster analysis (supplementary figure 7B), but also the vast majority of biopsy samples were separated according to the histological classification of the pathologist (κ_uncertain_=0.69, κ_overall_=0.89; supplementary figure 7C).

A gene ontology (GO) analysis of these 192 genes revealed a significant enrichment of genes involved in biological processes contributing to embryonic and organ morphogenesis (e.g. GO:0048562, FDR<1.53x10^-8^, enrichment: 12.52), development (e.g. GO:0007389, 4.76x10^-8^, enrichment: 6.15) and transcriptional regulation (e.g. GO:0006357, FDR<9.02x10^-9^, enrichment: 2.7) (supplementary table 6). These results are supported by gene ontology function analysis also indicating a significant enrichment of RNA polymerase II transcription factor activity (e.g. GO:0000981, FDR<7.21x10^-17^, enrichment: 4.71, supplementary table 7). A db-string analysis revealed interaction among numerous of these 192 genes, in particular MEIS1, PAX- and HOX-genes and components of the PI-signaling and responding cascade (supplementary table 8 and supplementary figure 9). In a subsequent analysis we focused on gene ontology terms unique for either the 1,303 loci aberrantly methylated in bronchoscopic biopsies or for the 10.098 loci aberrantly methylated in surgically resected primary tumors. It turned out that in the first GO-terms related to metabolic processes are to the prior while GO-terms related to G protein-coupled receptor signaling, cell communication/ cell junction, ion/ transmembrane transport as well as cell motility and migration are more frequent in or unique to the latter.

Next, we applied a t-test statistics (FDR<1x10^6^, delta.beta>0.25) to the data set of surgical specimens to identify loci differentially methylated between AC and SQC. A hierarchical cluster analysis of the DNA methylation values of the 1,112 resulting loci separated both entities (supplementary figure 10A). However, the set of 1,112 loci failed to separate the AC and SQC samples of the biopsy cohort (supplementary figure 10B).

These results suggest that DMA results and loci panels developed on a specific type of sample material should be transferred only with care to other types of sample materials with different characteristics or origin (i.e. biopsy and surgical specimens). At least in our hands, loci panels based on biopsy methylation data performed more reliably on the corresponding data sets of surgical specimens than vice versa. This might has to be taken into account when developing epigenetic biomarkers for clinical use in future.

In the analyses detailed above we performed a DMA on well characterized surgical specimens with high tumor cell content. Applying the same tests and thresholds for the DMA (FDR<1x10^-6^, delta.beta>0.25) of biopsies and surgical specimens resulted in 1,303 and 10,098 loci, respectively. Surprisingly, interception of these loci resulted in only 502 loci. This implies, that more than 60% of the loci (n=801) identified in the biopsy cohort have not been identified by the approach to analyze the cohort of surgical specimens. Nevertheless, these 502 loci differentiated malignant and non-malignant specimens in both cohorts. Again, also the two cases erroneously considered benign (P05140125T and P10130074T) in the histological evaluation, clustered with tumor samples. Gene ontology analyses of these genes revealed significant enrichment of processes and functions related to gene regulation and development (i.e. GO:0006357: regulation of transcription by RNA polymerase II (1.53x10^-8^) or GO:0048562: embryonic organ morphogenesis (FDR<1.53x10^-8^), please see supplementary table 6 for details), which is expected since altered gene regulation is a hallmark of cancer [15]. Major nodes and associations identified by a subsequently performed db-string analysis (supplementary table 8) revealed interactions between numerous factors involved in differentiation, development and gene regulation. PAX9 and PAX6 are members of the paired box family of transcription factors playing important roles during fetal development and cancer growth [16]. Homeobox genes are temporally expressed during embryonic development. HOX genes e.g. encode transcription factors regulating gene expression, morphogenesis, and differentiation [17]. MEIS1 also belongs to the homeobox proteins. It regulates cell proliferation in non-small cell lung cancer cells and has already been suggested as biomarker in lung cancer [18, 19]. Like MEIS1, EN1 and SIX3 also belongs to the homeobox proteins essential for development. Hypermethylation of EN1 has been shown in lung cancer in response to air pollution [20] while expression levels of SIX3 have been associated with the clinical outcome of AC [21]. TBR1 is a member of the T-box genes which encode for transcription factors particularly involved in the regulation of developmental processes [22]. The second cluster of interacting proteins contains e.g. TRIO, which promotes the reorganization of the actin cytoskeleton and is thought to play a role in cell migration, TAC1, which has already been suggested as biomarker in lung cancer [23], LPAR5, a G protein-coupled receptor, which affects tumor invasion and metastasis [24], WDFY2, which has been identified as a putative therapeutic target in e.g. prostate cancer [25] as well as PIK3CA, which encodes the catalytic domain of phosphatidylinositol 3-kinase and which has been functionally associated with NSCLC [26, 27].

Nevertheless, the majority of loci aberrantly methylated in the biopsy or resection cohort was unique to the respective sample cohort. This is also reflected in differences of the corresponding affected GO-terms. Biopsies collected during bronchoscopy, which were subsequently not further enriched for tumor cells contain probably much more non-malignant cells present in the tumor microenvironment as compared to the macrodissected surgical specimens of resected tumors. The resulting focus on malignant cells might explain most of the methylation differences in the sample cohorts. Additionally, tumor cells actively induce changes in neighbored cells [28]. These bystander effects might also contribute to an overall tumor specific methylation pattern, which can be useful for diagnostic purposes but which is separate from the pattern of enriched malignant cells, further explaining the differences in the methylation pattern of our cohorts, as these effects are hidden after tumor cell enrichment.

A more detailed analysis of the 801 aberrantly methylated CpG loci identified uniquely in the biopsy but not in the surgical specimens revealed a quite homogeneous DNA methylation pattern in all tumor samples, independently of whether they derived from the biopsy or surgical cohort (supplementary figure 11A/B). In contrast, the DNA methylation pattern of these 801 loci in the non-malignant samples showed clear differences in both cohorts. A t-test revealed that 639 of the 801 loci (>79%; corresponding to 352 individual genes) were differentially methylated between non-malignant samples of the biopsy and the surgical cohort (FDR<0.05; t-test, Bonferroni correction). In turn, only 5 loci (<0.7%) were differentially methylated between the tumor samples of the two cohorts. Also the medians of the absolute differences of the avg.beta-values of the 801 loci in both cohorts differed between the tumor (3.3%) and non-malignant samples (15.7%). Since the non-malignant biopsy samples were taken from the contralateral bronchi, while the distance between malignant and non-malignant areas of the surgical specimens was much smaller (>1cm), the differences between non-malignant biopsy samples and surgical specimens might most likely reflect an effect of the tumor cells on the surrounding normal tissue. As the tumor specimens of both cohorts presented very homogeneously, this observation is probably not related to tumor cell enrichment by macrodissection of the malignant cells in the surgical cohort. Albeit artefacts due to surgical intervention, anesthesia or hemostasis cannot be excluded.

Finally, one has to take into account that due to tumor heterogeneity tumor cells reachable during bronchoscopy and located proximal to the biopsied bronchus might show different methylation patterns as compared to more distal or central tumor regions available from resected tumor specimens.

Interestingly, in our data set all panels of differentially methylated loci as well as classifiers built on the biopsy samples performed well to identify the appropriate sample groups when applied to the DNA methylation data collected from surgical specimens (κ=1). In contrast, panels and classifier built on surgical specimens performed generally less to reliably separate the appropriate groups in the biopsy sample cohort. Although speculative the altered DNA methylation pattern might reveal a more general and overall tumor related pattern while after tumor cell enrichment of resected samples characteristics of the malignant cells might get in to the forefront and general effects become masked.

Nevertheless, these results also show that conclusions drawn from tumor cell enriched malignancies should only be transferred with care to other types of sample cohorts as our data indicate that these are probably two unique and distinct analytes, even if sharing many molecular and cellular characteristics.

**References**

1. Fane M, Harris L, Smith AG, Piper M. Nuclear factor one transcription factors as epigenetic regulators in cancer. Int J Cancer.;140(12):2634-2641.
2. Rahman NIA, Abdul Murad NA, Mollah MM, Jamal R, Harun R. NFIX as a Master Regulator for Lung Cancer Progression. Front Pharmacol.;8:540.
3. Freeman JR, Chu S, Hsu T, Huang YT. Epigenome-wide association study of smoking and DNA methylation in non-small cell lung neoplasms. Oncotarget. 2016;7(43):69579-69591.
4. Yuan C, Hu H, Kuang M, Chen Z, Tao X, Fang S, at al. Super enhancer associated RAI14 is a new potential biomarker in lung adenocarcinoma. Oncotarget. 2017;8(62):105251-105261.
5. Hsu YC, Chen HY, Yuan S, Yu SL, Lin CH, Wu G, at al. Genome-wide analysis of three-way interplay among gene expression, cancer cell invasion and anti-cancer compound sensitivity. BMC Med. 2013;11:106.
6. Jacobsen NR, Raaschou-Nielsen O, Nexø B, Wallin H, Overvad K, Tjønneland A, et al. XRCC3 polymorphisms and risk of lung cancer. Cancer Lett. 200;213(1):67-72.
7. Qiu M, Xu L, Yang X, Ding X, Hu J, Jiang F, et al. XRCC3 Thr241Met is associated with response to platinum-based chemotherapy but not survival in advanced non-small cell lung cancer. PLoS One. 2013;8(10):e77005.
8. Liu HX, Li J, Ye BG. Correlation between gene polymorphisms of CYP1A1, GSTP1, ERCC2, XRCC1, and XRCC3 and susceptibility to lung cancer. Genet Mol Res. 2016;15(4).
9. Rieke DT, Ochsenreither S, Klinghammer K, Seiwert TY, Klauschen F, Tinhofer I, et al. Methylation of RAD51B, XRCC3 and other homologous recombination genes is associated with expression of immune checkpoints and an inflammatory signature in squamous cell carcinoma of the head and neck, lung and cervix. Oncotarget. 2016;7(46):75379-75393.
10. Tang H, Wei P, Duell EJ, Risch HA, Olson SH, Bueno-de-Mesquita HB, et al. Axonal guidance signaling pathway interacting with smoking in modifying the risk of pancreatic cancer: a gene- and pathway-based interaction analysis of GWAS data. Carcinogenesis. 2014;35(5):1039-45.
11. Zhang B, Zhang Y, Zou X, Chan AW, Zhang R, Lee TK, et al. The CCCTC-binding factor (CTCF)-forkhead box protein M1 axis regulates tumour growth and metastasis in hepatocellular carcinoma. J Pathol. 2017;243(4):418-430.
12. Dallüge KH, Ziegenbein R. Enzymatic tests in the prognosis and course controls in bronchial carcinoma patients. Arch Geschwulstforsch. 1982;52(3):199-202.
13. Zhou H, Lv Q, Guo Z. Transcriptomic signature predicts the distant relapse in patients with ER+ breast cancer treated with tamoxifen for five years. Mol Med Rep. 2018;17(2):3152-3157.
14. Ramalingam A, Duhadaway JB, Sutanto-Ward E, Wang Y, Dinchuk J, Huang M, et al. Bin3 deletion causes cataracts and increased susceptibility to lymphoma during aging. Cancer Res. 2008;68(6):1683-90.
15. Hanahan D, Weinberg RA. Hallmarks of cancer: the next generation. Cell. 2011;144(5):646-74.
16. Lee JC, Sharma M, Lee YH, Lee NH, Kim SY, Yun JS, et al. Pax9 mediated cell survival in oral squamous carcinoma cell enhanced by c-myb. Cell Biochem Funct. 2008;26(8):892-9.
17. Zheng C, Jin FQ, Chalfie M. Hox Proteins Act as Transcriptional Guarantors to Ensure Terminal Differentiation. Cell Rep. 2015;13(7):1343-1352.
18. Li B, Lu Q, Song ZG, Yang L, Jin H, Li ZG, et al. Functional analysis of DNA methylation in lung cancer. Eur Rev Med Pharmacol Sci. 2013;17(9):1191-7.
19. Rauch TA, Wang Z, Wu X, Kernstine KH, Riggs AD, Pfeifer GP. DNA methylation biomarkers for lung cancer. Tumour Biol. 2012;33(2):287-96.
20. Jiang H, Deng R, Yang X, Shang J, Lu S, Zhao Y, et al. Peptidomimetic inhibitors of APC-Asef interaction block colorectal cancer migration. Nat Chem Biol. 2017;13(9):994-1001.
21. Mo ML, Okamoto J, Chen Z, Hirata T, Mikami I, Bosco-Clément G, et al. Down-regulation of SIX3 is associated with clinical outcome in lung adenocarcinoma. PLoS One. 2013;8(8):e71816.
22. Hevner RF, Shi L, Justice N, Hsueh Y, Sheng M, Smiga S, et al. Tbr1 regulates differentiation of the preplate and layer 6. Neuron. 2001;29(2):353-66.
23. Wrangle J, Machida EO, Danilova L, Hulbert A, Franco N, Zhang W, et al. Functional identification of cancer-specific methylation of CDO1, HOXA9, and TAC1 for the diagnosis of lung cancer. Clin Cancer Res. 2014;20(7):1856-64.
24. Lee SC, Fujiwara Y, Liu J, Yue J, Shimizu Y, Norman DD, et al. Autotaxin and LPA1 and LPA5 receptors exert disparate functions in tumor cells versus the host tissue microenvironment in melanoma invasion and metastasis. Mol Cancer Res. 2015;13(1):174-85.
25. Wang J, Chen X, Tong S, Zhou H, Sun J, Gou Y, et al. Overexpression of WDFY2 inhibits prostate cancer cell growth and migration via inactivation of Akt pathway. Tumour Biol. 2017;39(6):1010428317704821.
26. Trejo CL, Green S, Marsh V, Collisson EA, Iezza G, Phillips WA, et al. Mutationally activated PIK3CA(H1047R) cooperates with BRAF(V600E) to promote lung cancer progression. Cancer Res. 2013;73(21):6448-61.
27. Scheffler M, Bos M, Gardizi M, König K, Michels S, Fassunke J, et al. PIK3CA mutations in non-small cell lung cancer (NSCLC): genetic heterogeneity, prognostic impact and incidence of prior malignancies. Oncotarget. 2015;6(2):1315-26.
28. Florl AR, Steinhoff C, Müller M, Seifert HH, Hader C, Engers R, et al. Coordinate hypermethylation at specific genes in prostate carcinoma precedes LINE-1 hypomethylation. Br J Cancer. 2004;91(5):985-94.

**Figure legends**

**Supplementary Figure 5: A classifier separating benign and malignant samples.** Three independent approaches (KNN, SVM and RDT) to build a classifier separating benign and malignant biopsy samples with certain diagnosis resulted in the same set of 10 CpG loci. After extracting DNA methylation values of these loci from all (**A**) biopsy samples and (**B**) surgical specimens a hierarchical cluster analysis was performed. Boxes on top of the heatmap: light green: control (certain diagnosis), dark green: control (uncertain diagnosis), red; tumor sample, certain diagnosis, orange: putative tumor sample, histological evaluation inconclusive. heatmap: blue: low, yellow: high DNA methylation. For presentation, mean methylation of each locus was normalized to zero (mean=0); heatmap: blue: low, yellow: high DNA methylation.

**Supplementary figure 6: Unsupervised principal component analysis (PCA) of DNA methylation data collected from surgical specimens** of 15 AC (red spheres), 19 SQC (blue spheres) and their corresponding controls (green spheres) using the HumanMethylation450 BeadChip. The complete, not further processed data set was used for PCA.

**Supplementary figure 7:** (**A**) 10,098 loci differentially methylated between tumor containing surgical specimen (n=40) and their corresponding normal controls were subjected to hierarchical cluster analysis (Wilcoxon paired test, FDR<1x10^-6^, delta.beta>0.25). As a result tumor (red boxes) and control specimens (green boxes) were separated. (**B**) (**C**) Interception of loci aberrantly methylated in tumor samples of reliably diagnosed paired biopsies and surgical tumor specimens resulted in 502 loci. These loci were extracted from (**B**) surgical specimens or (**C**) all biopsy specimens and the corresponding DNA methylation data were subjected to hierarchical cluster analysis. heatmap: blue: low, yellow: high DNA methylation. For presentation, mean methylation of each locus was normalized to zero (mean=0).

**Supplementary figure 8:** 10.098 loci differentially methylated between tumor specimens and control samples in the cohort of surgically resected specimens were extracted from the data set of the biopsy cohort. Subsequently a hierarchical cluster analysis was performed. Boxes on top of the heatmap: red: tumor samples, green: controls, heatmap: blue: low (avg.beta=0), yellow: high (avg.beta=1) DNA methylation.

**Supplementary figure 9:** 192 genes found aberrantly methylated in malignant samples of both, surgical as well as biopsy specimens were subjected to string-db analysis.

**Supplementary figure 10:** A t-test analysis revealed 1,112 loci differentially methylated between AC and SQC in the cohort consisting of surgical specimens (FDR<1x10^-5^, σ/σ_max_>0.4). (**A**) Hierarchical cluster analysis of the DNA methylation values of these loci in the cohort of surgical specimens. Boxes on top of the heatmap: blue: SQC samples, red: AC specimens. (**B**) Hierarchical cluster analysis of these 1,112 loci in the biopsy cohort. Boxes on top of the heatmap: upper lane; green: control samples, red: tumor samples. Lower lane: yellow: SQC, pink: AC. heatmaps: blue: low, yellow: high DNA methylation. For presentation, mean methylation of each locus was normalized to zero (mean=0). The bar below indicates the DNA methylation state (relative to the mean).

**Supplementary figure 11: (A) Hierarchical cluster analyses and (B) PCA of 801 CpG loci differentially methylated between non-malignant lung tissue samples and lung cancer specimens in the bronchoscopic biopsy cohort but not in the cohort of surgically resected tissues.** Red boxes on top of the heatmap: lung carcinoma samples, light green boxes: non-malignant lung tissue samples, blue boxes: brochoscopic biopsy samples, yellow boxes: surgical specimens. Heatmap: blue: low avg.beta values, yellow: high avg.beta-values.

**Supplementary tables**

**Supplementary table 5:** Gene ontology analysis (Gorilla tool; biological process) of aberrantly methylated genes in surgically resected specimens. Genes present on the array and included in the study acted as background list.

**Supplementary table 6:** Gene ontology analysis (Gorilla tool; biological process) of 192 genes commonly aberrantly methylated in the tumor samples of both, surgical and biopsy specimens. Genes present on the array and included in the study acted as background list.

**Supplementary table 7:** Gene ontology analysis (Gorilla tool; biological function) of 192 genes commonly aberrantly methylated in the tumor samples of both, surgical and biopsy specimens. Genes present on the array and included in the study acted as background list.

**Supplementary table 8:** String-db analysis of 192 genes commonly aberrantly methylated in the tumor samples of both, surgical and biopsy specimens. See also supplementary figure 9.
